# Expert consensus on the procedure of interventional diagnosis and treatment of cancer patients during the COVID-19 epidemic

**DOI:** 10.1016/j.jimed.2020.03.001

**Published:** 2020-03-31

**Authors:** Interventional Oncology Branch of China Anti-Cancer Association, Tianshi Lyu, Li Song, Long Jin, Yinghua Zou

**Affiliations:** a Department of Interventional Radiology and Vascular Surgery, Peking University First Hospital, Beijing, 100034, China; b Department of Interventional Radiology, Beijing Friendship Hospital, Capital Medical University, Beijing, 100050, China

**Keywords:** Novel coronavirus, Pneumonia, Intervention, Expert consensus

## Abstract

Since December 2019, coronavirus disease (COVID-19) has spread rapidly from Wuhan, Hubei province, to other regions of China. To reduce and prevent cross-over infections in the interventional diagnosis and treatment of tumor patients. The Interventional Oncology Branch of the China Anti-Cancer Association organized specialists to compile the corresponding expert consensus. The consensus summarizes the critical points for COVID-19 prevention, focusing on the management of outpatients, inpatients, and interventional operating room in this particular time.

Since December 2019, the Hubei province, especially Wuhan, has successively diagnosed multiple cases of the new-type coronavirus pneumonia. With the spread of the virus, similar cases have also been found in other provinces of China and even abroad.^1^ On February 11, 2020, the International Committee on Taxonomy of Viruses announced that the official classification of the new coronavirus (2019-nCoV) was severe acute respiratory syndrome coronavirus 2, SARS-CoV-2); while on that same day, the World Health Organization announced that the official name of the disease was COVID-19.

On February 17, the National Health Commission issued a report to guide local governments to strengthen medical service management amid the epidemic, maintain the routine medical order, and meet the basic medical needs of the people. According to the announcement, all areas should maintain routine medical order based on scientific prevention and epidemic control. Medical institutions should perform classified treatments according to the different patients to meet their medical needs.^2^

Patients diagnosed with malignancies are undoubtedly severely affected by COVID-19. Although the time of the surgical procedure can be selected, there is a specific limit, and treatment, such as chemotherapy or embolization of malignant tumors, should not be delayed too long. In addition, in some emergency situations, such as tumor bleeding, surgery should be performed immediately to save the patients’ lives. The Interventional Oncology Branch of the China Anti-Cancer Association formed a panel of experts to sort out and summarize the process of interventional diagnosis and treatment of cancer patients. They wrote the expert consensus, hoping that the anti-epidemic and interventional tumor treatment will be coordinated and implemented in an orderly manner. Each region can execute this consensus based on their actual situation.

## 1 General principles^2^

### 1.1 Screen first, to identify suspected cases as early as possible

Besides reviewing symptoms and vital signs (especially body temperature), COVID-19 epidemiological surveys should also be conducted on all patients in interventional clinics, wards, and operating rooms. Before admission, a chest computerized tomography (CT) and blood tests are recommended. New coronavirus nucleic acid tests should also be performed for screening, if applicable. For the suspicious cases identified in the screening process, the corresponding process should be started immediately, which includes isolation and report to a superior department in the hospital. In addition, the contact, droplet and airborne should all be isolated as recommended.

### 1.2 Protect the medical needs according to the criticality and severity of the tumors

For critically ill patients, timely and effective treatment should be given following relevant regulations and guidelines. Consider ensuring performing emergency interventional surgeries, patients with rapid tumor progression or urgent treatment needs should be admitted, and surgeries performed in a timely manner following proper infection prevention and control. For patients with elective surgeries, it is paramount to strengthen the communication with patients; elective surgeries should be arranged in an orderly manner according to both the epidemic and patients’ conditions.

### 1.3 Implement rigorous standard prevention measures, focused on wearing surgical face masks and hand hygiene to avoid nosocomial infections

Standard prevention should treat all patients as infectious patients to prevent the spread of both blood-borne and non-blood-borne diseases, to emphasize two-way protection between patients, companion, and medical staff. In addition to standard prevention, corresponding isolation measures should be taken according to the main transmission route of the disease, including contact, droplet, and airborne isolation. All staff, patients, and their companions in interventional clinics, wards, and operating rooms should wear surgical face masks and perform proper hand hygiene.

## 2 Management of infection prevention and control in interventional clinics

2.1 Outpatients must make appointments by telephone or other online ways in advance before go to hospitals. Before entering the outpatient area, patients and companions should have their temperature taken, undergo an epidemiological and symptomatic screening, and sign a legally-binding consent at the triage office. Suspicious cases will be referred to the nearest fever clinic. Patients and companions must wear surgical face masks correctly in the hospital areas at all times.

2.2 Interventional radiology personnel should strictly follow the standard precautionary principles, with particular importance to personal protection, hand hygiene, ward management, environmental ventilation, disinfection of equipment, and medical waste management, etc. These measures may prevent nosocomial infections substantially.^3^ All interventional radiology personnel should wear surgical face masks during the diagnosis and treatment activities, strictly follow the outpatient consultation principle of “one room, one doctor, and one patient,” and minimize the staff in the consultation room. A certain distance must be kept between doctors, and patients during the consultation and close contact must be avoided as much as possible.

2.3 Relevant medical personnel in triage offices should wear medical suits, surgical face masks, and surgical caps.

2.4 Oncology patients presenting a fever should go to the fever clinic designated by the local health department first. After COVID-19 is ruled out, the patient can go to the interventional outpatient clinic for treatment.

## 3 Principles of epidemic prevention and control in interventional wards

### 3.1 Screening of newly admitted patients

Before admission, patients should complete a pre-screening process in the outpatient clinic. Routine chest CT and blood tests are recommended. New coronavirus nucleic acid tests should be performed at the same time, if applicable. Strictly follow the established admission principles. Patients can request admission to the hospital and sign a legally-binding commitment letter after COVID-19 is ruled out. The attending physician will decide whether the patient can be admitted according to his or her general condition and body temperature.

### 3.2 Patient management

Meals delivery services from outside of the hospital are prohibited, the meals should be contained in disposable containers and delivered by nursing staff. Family visits are prohibited as well. For those who require caregivers or companions due to their critical condition, this person cannot be replaced by another during the hospitalization period and must undergo the same screening process in conjunction with the same daily temperature monitoring as the patients. In principle, new admission is not allowed into the same room on the day of discharge, the whole room should be properly cleaned and disinfected. As a result, the new patient should be admitted the next day.

Wards are strictly managed at all times. In the following cases, entry to the ward is prohibited:

1 Fever (body temperature ​≥ ​37.3 ​°C) and COVID-19 has not been ruled out in the fever clinic, or there are other suspicious symptoms, such as a cough and fatigue.
2 History of residence or travel within the past 14 days to the Hubei province or surrounding areas;
3 History of residence or recent travel to communities with reported cases of COVID-19 within the past 14 days;
4 Exposure to infected patients diagnosed with COVID-19 within the past 14 days, or suspicious patients;
5 Two or more family members or coworkers presented a fever or respiratory symptoms within the past 14 days.

If someone is found to have fever during hospitalization, the case should be notified to the fever clinic for an immediate consultation. If COVID-19 is ruled out in the person with fever, routine processes for diagnosis and treatment should be carried out. If the person with fever is suspicious of COVID-19 infection, the corresponding process flow should start immediately, and the case should be reported to the hospital administration. The patient should be transferred to the isolation ward or designated hospital as soon as possible. The ward should be strictly and entirely disinfected. In addition, anyone who had close contact with this person should be under surveillance isolation as well.

### 3.3 Personal protection for medical personnel^3^

Standard preventive measures, such as general ward ventilation management, hand hygiene, and correct usage of surgical face masks and caps, should be vigorously implemented. Latex gloves should be worn if necessary. Adequate protective measures for droplet, contact, and airborne isolation should be stressed. Personal protection may vary on a case-by-case basis. The consultation area should be well ventilated, with daily cleaning, and final disinfection.

### 3.4 Other recommendations

It is recommended to set up an isolation unit within the interventional ward. If a patient is suspected of having COVID-19, he/she should be transferred to this isolation unit immediately.

It is recommended that the total number of patients should not exceed half of the usual number. Admitted patients are advised to have one room per person or sufficient isolation space to reduce the possibility of cross-contact.

It is recommended to adjust the ward rounds system during this particular time period; only attending physicians will attend the ward rounds.

## 4 Principles of epidemic prevention and control in the interventional operating room^4^, ^5^, ^6^

### 4.1 Routine interventional operations

#### 4.1.1 Patients screening

1 Normal body temperature or, although fever was detected, COVID-19 infection has been ruled out by the fever clinic.
2 Negative results in epidemiological and symptomatic screening and signed written commitment.
3 Recent chest CT examination does not meet the criteria for viral pneumonia, or the patient has negative new coronavirus nucleic acid test results.

#### 4.1.2 Surgery

1 Use disposable surgical kits, surgical instruments, auxiliary materials, consumables, etc. During the surgery, have available the required items for the procedure as much as possible to reduce personnel entry and exit.
2 After every surgery, one-time protective items should be changed, ECG monitoring kits should be disinfected. Other items, such as infusion racks, ECG monitors, high-pressure syringes, ventilators, etc., need to be covered by disposable plastic films.
3 Place all disposable items in the yellow garbage bag and keep it tightly closed.
4 After surgery, medical waste sorting and surrounding environment disinfection should be implemented.
5 Before proceeding to the next surgery, use ultraviolet light disinfection for about 15–20 ​min.

#### 4.1.3 Personal protection for medical staff

1 Complete personal protection by standard protection protocols should be vigorously implemented.
2 In addition to routine preparations, surgeons are recommended to wear goggles or surgical face masks.
3 Limit the number of people in the operating room.
4 Follow the best practices to prevent occupational exposure.

### 4.2 Emergency interventional operations

Patients who need emergency surgeries but cannot meet the above screening conditions are considered as suspected cases of COVID-19.

### 4.3 Operations for confirmed or suspicious infected patients

#### 4.3.1 Preoperative management

4.3.1.1 After a comprehensive assessment of the patient’s overall condition and COVID-19 status, a multidisciplinary expert team and interventional radiologists should come to a consensus as to whether or not this patient should undergo emergency interventional surgery.

4.3.1.2 Informed consent should be signed by family members who have no history of close contact with the patient. If the family members have a close contact history and are under surveillance isolation, surgeons can communicate with them by telephone and should document these interventions. If the patient doesn’t have any family members, report it to the medical office for the record.

4.3.1.3 Hospitals should set up a green channel for preoperative transportation to speed up the transport rate and reduce cross-infection. After the green channel is used, it should be disinfected with a solution containing at least 1000 ​mg/L of chlorine. Staff involved in transportation should wear level-two protective equipment.^3^^,^^7^

4.3.1.4 A special interventional operating room should be set up, and a negative pressure operating room should be used if possible. Centralized air conditioning should be turned off.

4.3.1.5 To limit the frequent personnel entry and exit, prepare the necessary instruments, consumables, and medicines as much as possible for the type of surgery. The items can only be taken into the operating room rather than out. All item surfaces should be covered with double layers of disposable plastic films, which include surgical instruments, accessories, consumables, surgical beds, instrument tables, etc. Disposable plastic films should also cover other items, such as infusion racks, ECG monitors, high-pressure syringes, ventilators, etc.

#### 4.3.2 Intraoperative management

4.3.2.1 Limit the number of people involved in the operating room. Medical staff with open wounds should not participate in the surgery. The circulating nurse cannot leave the operating room.

4.3.2.2 Pay attention to the protection of the surgeons. The surgeon and the anesthesiologist should wear level-three protective equipment with an extra pair of sterile gloves.

4.3.2.3 The disinfection of the operating room should be carried out in strict accordance with relevant regulations and best practices. Before and after surgery, the floors should be disinfected routinely with a 1000 ​mg/L chlorine-containing disinfectant. If there is any fluid or blood left on the floor or the surfaces of items, it should be wiped immediately with 2000 ​mg/L chlorine-containing disinfectant.

4.3.2.4 After the operation, the personnel involved in that surgery should remove their personal protective equipment in the designated isolation area outside the operating room, per guidelines and regulations. Hand disinfection should be performed at the end of each step of removing the protective equipment, and it also should be performed again after all protective equipment has been removed.

#### 4.3.3 Postoperative management

4.3.3.1 The patient should be transferred to the designated isolation ward after the surgery to complete the required treatments.

4.3.3.2 The janitors must wear isolation suits, disposable hats, N95 masks, and disposable gloves to disinfect and clean the surfaces of items and the floors of the operating room. In addition, air disinfection should be carried out as well.

4.3.3.3 All medical waste should be transported in a sealed double-layer medical garbage bag and marked with the special identification of “COVID-19,” which will be properly treated according to strict regulations.

4.3.3.4 The infection control department must perform sampling of the surfaces and the air after proper disinfection of the surgical room. The operating room can be used again only if the sampling test is passed.

## 5 Final words

Although the new COVID-19 pneumonia epidemic tends to curb, health care professionals should remain alert to prevent cross-infection while treating oncology patients. It is hoped that all health care professionals will fight side by side with our colleagues and work together to win the battle against both the COVID-19 epidemic and cancer.

## Declaration on interests

The authors declare that they have no known competing financial interests or personal relationships that could have appeared to influence the work reported in this week.
